# Multi-objective-conditioned-return-blocks decision transformer: a novel approach for multi-objective *de novo* drug design

**DOI:** 10.1093/bioinformatics/btag731

**Published:** 2026-10-01

**Authors:** Eyal Mazuz, Or Gindes, Lior Rokach, Bracha Shapira

**Affiliations:** Faculty of Computer and Information Sciences, Ben-Gurion University of the Negev, Be'er Sheva 8410501, Israel; Faculty of Computer and Information Sciences, Ben-Gurion University of the Negev, Be'er Sheva 8410501, Israel; Faculty of Computer and Information Sciences, Ben-Gurion University of the Negev, Be'er Sheva 8410501, Israel; Faculty of Computer and Information Sciences, Ben-Gurion University of the Negev, Be'er Sheva 8410501, Israel

## Abstract

**Motivation:**

Designing novel molecules that simultaneously satisfy several, often conflicting, properties is a long-standing challenge in drug design. While recent deep learning advances optimize single targets effectively, navigating the tradeoffs required for multiple objectives remains a difficult problem.

**Results:**

We introduce the Multi-Objective-Conditioned-Return-Blocks Decision Transformer (M-GoRT), a novel architecture for multi-target de-novo drug design incorporating return-to-go blocks conditioned on goal embeddings. Trained without exploration, reward weighting or online fine-tuning, M-GoRT efficiently optimizes for multiple targets. M-GoRT achieves competitive multi-objective success rates against state-of-the-art baselines while maintaining superior structural diversity and novelty, even in high-dimensional tasks incorporating biological targets (DRD2, JNK3). In these high-dimensional tasks, M-GoRT maintains superior structural diversity and novelty establishing it as a practical tool for multi-property molecular generation.

**Availability and implementation:**

M-GoRT and the data used are freely available as an open-source software under the MIT license at GitHub https://github.com/eyalmazuz/MolGen/tree/GCRB-DT.

## 1 Introduction

A major challenge in pharmaceutical development is the time-consuming and resource-intensive process of *de novo* drug design (Mouchlis *et al*. 2021, Bagal *et al.* 2022). Traditional approaches rely on labor-intensive synthesize-and-test cycles conducted by pharmacologists, who optimize for desired molecular attributes based on their expertise and domain knowledge (Fu and Chen 2025). The difficult task of designing molecules that satisfy multiple, yet conflicting objectives highlights the need for better multi-target molecular generation models (Nicolaou *et al.* 2007, Deng *et al*. 2022).

The recent revolution in generative artificial intelligence has opened unprecedented opportunities for accelerating molecular design (Jiménez-Luna *et al.* 2021, Zhang 2021). Deep generative models have demonstrated remarkable capabilities in learning complex molecular representations and generating novel compounds with desired properties, exploring vast chemical spaces more efficiently than traditional methods and potentially discovering molecular structures that human experts might overlook. While the application of generative AI to drug discovery has evolved from simple property optimization to sophisticated multi-objective design, existing computational methods often still focus on single-objective generation (Liu *et al.* 2021, Pang *et al.* 2024). Techniques for multi-objective optimization (MOO), such as evolutionary algorithms and reinforcement learning (RL), have been leveraged in some cases to manage the complex task of balancing competing optimization goals, nevertheless, effective multi-target molecular generation continues to pose substantial difficulties (Fromer and Coley 2023). Current approaches typically require either extensive online fine-tuning for each new combination of objectives, rely on carefully tuned scalarization weights that may not capture the true tradeoffs between objectives, or necessitate domain expertise to select appropriate molecular scaffolds as starting points (Luukkonen *et al.* 2023).

Despite significant advancements, balancing varied molecular objectives while maintaining structural validity and diversity remains challenging. Our proposed architecture, M-GoRT, introduces parameterized, goal-specific return-to-go (RTG) blocks conditioned by multiple goal embeddings that act as conditioning pathways, influencing the transformer’s architecture at every layer rather than using separate output heads. This extension to the standard RTG enables our model to simultaneously optimize generation for multiple molecular properties when trained using existing molecular datasets and without the need to condition generation on a few preset molecules, online fine-tuning, retraining for different objective preferences, or extensive post-processing. To the best of our knowledge, M-GoRT is the first application of a goal-conditioned Decision Transformer (DT) architecture for multi-objective molecular generation, offering enhanced flexibility and improved performance for complex multi-objective scenarios. Our main contributions are as follows:

1. Introducing the Multi-Objective-Conditioned-Return-Blocks Decision Transformer (M-GoRT), a DT-based architecture designed for optimized multi-objective molecule generation by utilizing goal-specific parameterized RTG blocks to condition the model.
2. Demonstrating that multiple goal-specific RTG blocks are essential for the model’s ability to optimize multiple goals simultaneously.
3. Evaluating our method’s performance using multiple metrics and comparing it with current state-of-the-art solutions for multi-objective molecular generation.

### 1.1 Related works

Molecular generation for drug discovery frequently requires optimizing multiple conflicting properties. While traditional Pareto-based methods and evolutionary algorithms (Luukkonen *et al.* 2023) can be computationally demanding, scalarization methods, which reduce targets to a single score via weighted sums, remain popular despite their reliance on rigid weight tuning and tendency to obscure implicit tradeoffs (You *et al.* 2018, Zhou *et al.* 2019, Xie *et al.* 2021, Wu *et al.* 2021, Suzuki *et al.* 2024). To mitigate these limitations, recent approaches incorporate Pareto-awareness into scalarization (Liu *et al.* 2021, Shin *et al.* 2025), sample diverse tradeoffs via preference-conditioning (Zhu *et al.* 2023b), or employ progressive and curriculum learning to dynamically adjust objective emphasis and manage the computational costs of expensive scoring oracles (Wang *et al.* 2021, Guo *et al.* 2022, Shin *et al.* 2024).

Beyond optimization strategies, generative architecture heavily impacts performance. While varied models such as VAEs, RNNs, GANs, and diffusion models have been extensively explored for both conditional and unconditional generation (Guimaraes *et al.* 2017, Jin *et al.* 2018, Fu *et al.* 2021, Shin *et al.* 2021, Wang *et al.* 2022, Yao *et al.* 2023, Shih *et al.* 2023, Alakhdar *et al.* 2024), Transformers have proven particularly adept at managing the long-range dependencies inherent in molecular representations (Mazuz *et al.* 2023, Loeffler *et al.* 2024). Building on this, DTs utilize RTG mechanisms to condition generation on expected cumulative rewards, effectively addressing sparse rewards in sequential tasks (Chen *et al.* 2021). Although recent developments demonstrate DTs’ efficacy in multi-objective RL (Bae *et al.* 2023, Zhu *et al.* 2023a), effectively balancing multiple objectives while preserving structural validity and chemical diversity remains challenging. Our proposed M-GoRT architecture addresses this limitation by introducing distinct, goal-specific RTG blocks for each objective, enabling the simultaneous optimization of conflicting properties without the need for online fine-tuning or retraining.

## 2 Materials and methods

In this section, we present the details of our proposed M-GoRT method, which combines the DT framework with multiple goal-conditioned RTG blocks to achieve effective multi-objective molecular generation.

### 2.1 Problem formulation

We define molecule generation and optimization as an autoregressive sequence-generation task where each sequence is an ordered list of tokenized elements. Concretely, we denote a trajectory τ as the sequence of tokens, where each token encodes a complete or partial molecular structure with its corresponding states s, actions a, and returns r. The trajectory is structured as:


$$\tau =\left\{\left(r^{1},\ldots ,r^{n},s_{1},a_{1}),\ldots ,(r^{1},\ldots ,r^{n},s_{T},a_{T}\right)\right\}$$

where $s_{t}$ is the generated tokenized SMILES string, $a_{t}$ is the next token generated at step t∈T, where *T* is the final length of the sequence. For each optimization goal i∈{1,…,n}, $r^{i}$ is the return target, which we set as the terminal property value (e.g. QED, pLogP) of the complete molecule in training or the desired property value in generation. The learning objective is to maximize the likelihood of the optimal action sequence given the prescribed goal-conditioned returns.

### 2.2 Overview

The backbone of the proposed method follows the standard DT pipeline: returns, states, and actions are all projected into a common *d*-dimensional latent space via modality-specific embedding layers. The resulting embeddings form an autoregressive token stream processed by a causally masked self-attention stack to predict the next action token (Chen *et al.* 2021). M-GoRT builds on this by replacing the single RTG embedding with a sequence of *n goals-conditioned return blocks*. At each step, the token tuple is


$$\left(e_{t}^{1},\ldots ,e_{t}^{n},\;S_{t},\;A_{t}\right),$$

where $e_{t}^{i}$ is produced by the *i*th return block and $S_{t},A_{t}$ are the state and action embeddings used in the canonical DT encoder. Figure 1 illustrates this process, highlighting the fusion of goal-conditioned return embeddings, current states, and previous actions to predict subsequent tokens.

**Figure 1:**
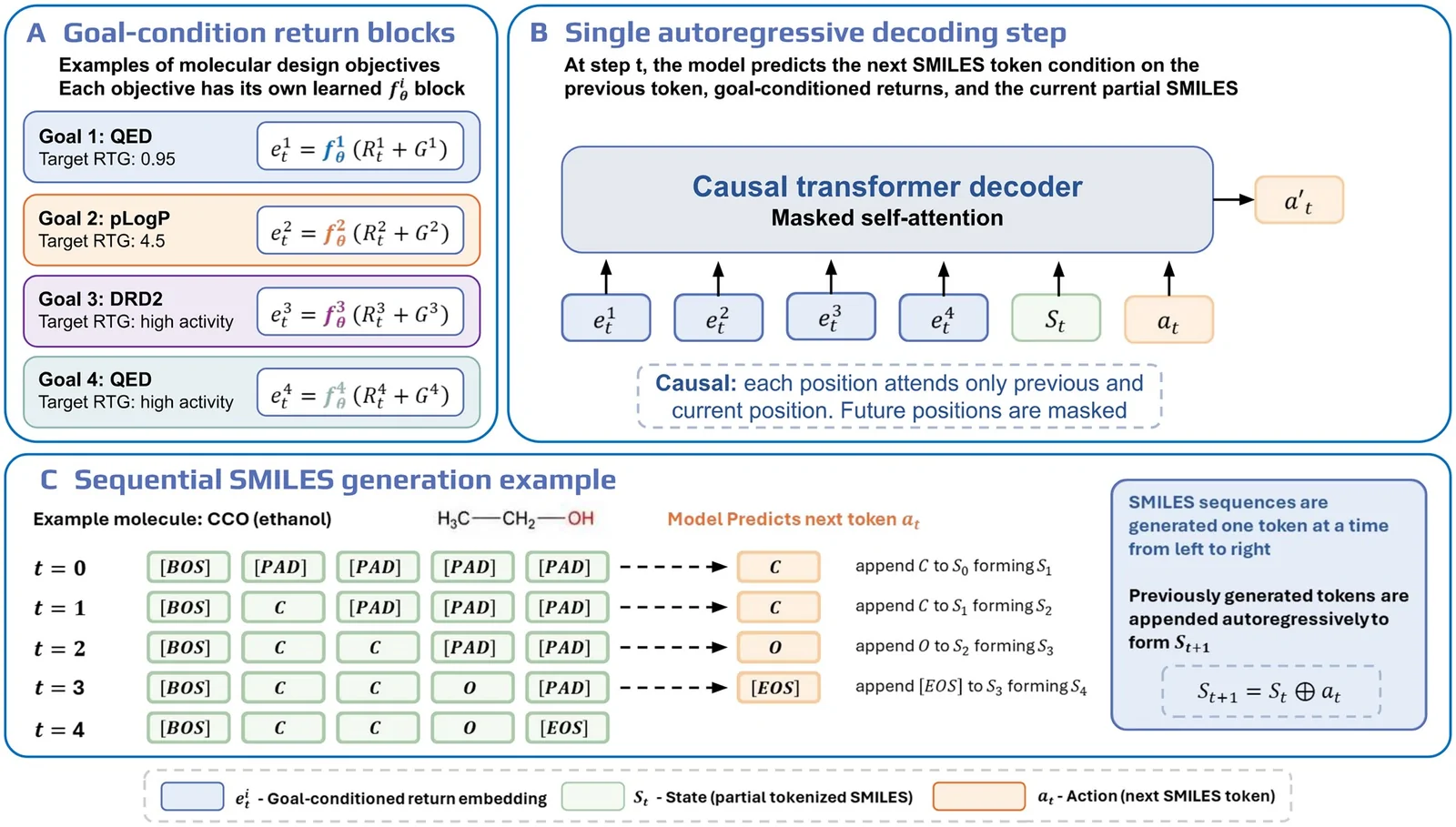
Multi-Objective-Conditioned-Return-Blocks (M-GoRT) decision transformer architecture for multi-objective molecular generation. The architecture consists of three key components: (A) Goal-conditioned return blocks: each molecular design objective (e.g. QED, pLogP, DRD2, or JNK3 bioactivity) uses a dedicated learned block, $f_{\theta }^{i}$, which combines the target return $R_{t}^{i}$ with a learned goal embedding $G^{i}$ to produce the goal-conditioned return embedding $e_{t}^{i}$. (B) Single autoregressive decoding step: the causal transformer decoder predicts the next action token $a_{t}^{\prime }$ from the previous action $a_{t-1}$, the goal-conditioned return embeddings $e_{t}^{i}$, and the current state $S_{t}$. Masked self-attention ensures that predictions depend only on past and current positions. (C) Sequential SMILES generation example: molecules are generated left-to-right, one SMILES token at a time. Using ethanol (CCO) as an illustration, at each step *t*, the predicted action $a_{t}$ is appended to the current partial tokenized SMILES state $S_{t}$ to form the subsequent state $S_{t+1}$, continuing until the [EOS] token is generated.

#### 2.2.1 States

Each trajectory state is represented as an n×n matrix, where *n* denotes the length of a tokenized SMILES sequence, augmented by special beginning-of-sequence ([BOS]) and end-of-sequence ([EOS]) tokens. A character-level tokenizer trained on SMILES molecules converts these strings into discrete token ID sequences. Each row *t* of the state matrix encodes a partial SMILES sequence, starting with the [BOS] token and extending up to the *t*th token, with subsequent indices filled by padding tokens ([PAD]). These padding tokens are masked during training to ensure the model only attends to the relevant tokens at each prediction step.

In practice, multiple states are processed simultaneously, forming input tensors with dimensions (batch_size,n,n), where *n* is the length of the longest sequence in the batch. Each slice (·,t,·) along the second dimension corresponds to all states at timestep *t*, providing partial sequences for batch predictions (illustrated in Fig. 2, available as supplementary data at *Bioinformatics* online). These matrices are transformed into continuous embeddings via a state embedding layer, followed by an attention-aware mean pooling operation that averages embeddings along each row while excluding padded tokens. This results in a compact, context-aware representation $S_{t}$ for each state.

**Figure 2:**
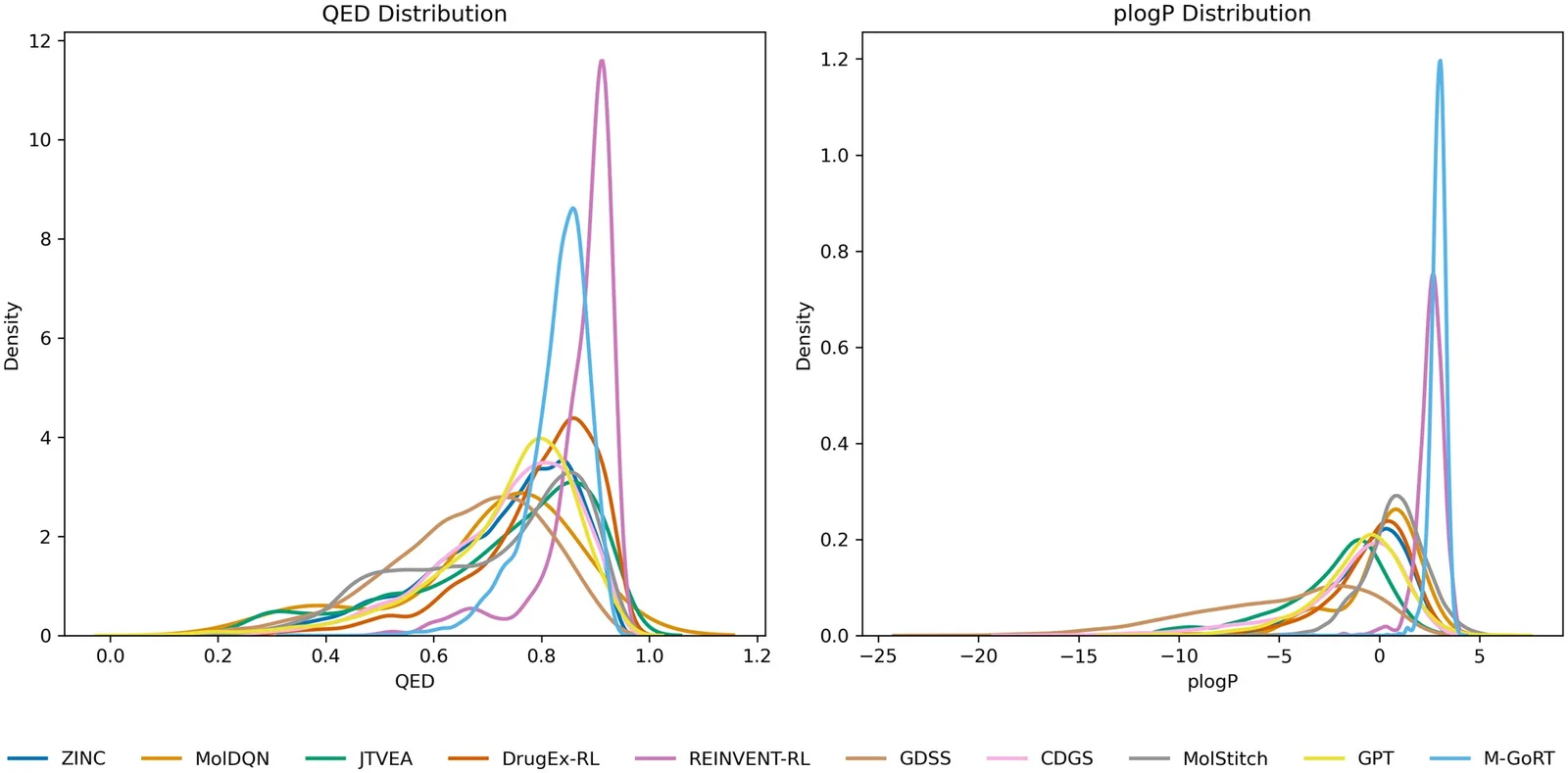
Distributions of QED (left) and pLogP (right) scores for valid and unique molecules found in ZINC and generated by baseline models and M-GoRT. The molecules from the ZINC dataset are shown in blue, and the molecules generated by MolDQN, JTVEA, DrugEx-RL, REINVENT-RL, GDSS, CDGS, MolStitch, GPT-o4-mini, and M-GoRT. Overall, REINVENT and M-GoRT are the only methods to visibly shift the distribution of both optimized properties compared to the ZINC dataset.

#### 2.2.2 Actions

The action sequence corresponding to each state is represented as a vector of length *n*, consisting of token IDs that sequentially build the SMILES sequence. This vector initiates with the first token ID following the [BOS] token and concludes with the [EOS] token ID. The actions vector is then passed through its dedicated embedding layer with the same dimensionality as the state embedding layer, producing the action embeddings $A_{t}$.

#### 2.2.3 Goal-conditioned returns

In standard DT architecture, the return represents the desired cumulative reward from the current step to the end of the trajectory. However, partial SMILES sequences can be chemically invalid, lacking meaningful returns. To address this, we maintain a constant desired return throughout the trajectory:


$$r_{t}^{i}=r_{\tau }^{i},\;t=1,\ldots ,T.$$

During training, each goal’s returns are a vector of length *n*, repeating the terminal molecule’s computed value at every timestep, paired with a corresponding goal index repeated across the same length.

Each optimization objective *i* utilizes a dedicated goal-conditioned return block, a two-layer MLP $f_{\theta }^{i}$ with ReLU activation:


$$e_{t}^{i}=f_{\theta }^{i}\left(R_{t}^{i}+G^{i}\right)\in R^{d_{\mathrm{model}}},$$

where $G^{i}$ is a learned goal embedding. Parameters are block-specific, enabling adaptation to distinct property distributions. These embeddings, together with state and action tokens, form the transformer’s input sequence. During generation, target property values ${\hat{R}}^{i}$ guide autoregressive token prediction until an [EOS] token or maximum length is reached. To enhance training stability, we adopt a curriculum style approach, beginning with a single objective and its return block and gradually activating the rest for joint multi-objective optimization.

#### 2.2.4 Molecule generation

The generation process begins with the initialization of the sequence using the [BOS] token, a target RTG values for each conditioning goal. The embeddings of the initial state, return signal, and goal indices pass through the transformer to generate the prediction logits from which token probabilities are calculated and the action token is sampled from to the resulting distribution.

Following the first action, tokens are continuously generated autoregressively, appending each newly sampled action to the state sequence for the next prediction so that the model’s input includes all previous states and actions taken in the trajectory. The process iterates until an end-of-sequence token [EOS] is produced or a maximum sequence length is reached.

### 2.3 Language model backbone

We adopt a GPT-style decoder-only transformer with *L* blocks, *h* attention heads, and hidden width $d_{\mathrm{model}}$. Each modality—constant RTG, state token, action token—has a learnable linear projection to $R^{d_{\mathrm{model}}}$. Tokens pass through causally masked multi-head self-attention and position-wise feed-forward layers as originally described by Vaswani *et al.* and adapted for DTs.

## 3 Results

To evaluate our model’s performance, we report the results on both single and multi-objective molecular optimization and generation tasks, treating single-target results as a baseline to quantify the performance tradeoffs of multi-objective optimization. We further compare M-GoRT performance to several SOTA baselines across multiple metrics. All of the models were trained on the ZINC dataset, which consists of 250K molecules (Irwin and Shoichet 2005].

### 3.1 Baselines

We benchmarked our model against several SOTA methods employing distinct optimization strategies: JTVAE (Jin *et al.* 2018) for junction-tree scaffold assembly, MolDQN (Zhou *et al.* 2019) for Deep Q-learning graph optimization, and diffusion-based methods like GDSS (Jo *et al.* 2022) and CDGS (Huang *et al.* 2023). We also evaluated RNN-based RL methods including DrugEx2 (Liu *et al.* 2021) and REINVENT (Olivecrona *et al.* 2017), as well as MolStitch (Shin *et al.* 2025), an offline framework utilizing a neural crossover operator. Finally, we included GPT-o4-mini, prompted for expert-chemist SMILES generation. For models requiring scalarization, we used a standard weighted sum of normalized objectives.

While our method does not relay on scalarization for multi-target optimization, some of these baselines do require a scalarized optimization goal for which we used the standard weighted sum method with equal weights for each goal and normalized objective values:


(1) $$R_{\text{total}}=\frac{1}{n}\sum_{i=1}^{n}R_{i}$$

All models were tested using publicly available code, their default hyperparameter values and on the same hardware containing one TITAN RTX GPU, 60 GB RAM, and an eight-core CPU.

### 3.2 Model configuration

For our experiments, our proposed method consists of a Transformer model with 16 decoder layers, each with 12 attention heads, an embedding size of 768, and block size of 300, resulting in a maximum sequence size of 100 characters. These parameters are similar to the ones used when the DT model was originally proposed (Chen *et al.* 2021) with the exception of the number of layers which has been tuned for improved performance; the remaining parameters have not been subject to optimization in this work. Each layer of the model includes a feed-forward network with dropout applied to the embeddings, attention weights, and residual connections at a rate of 0.1, to prevent overfitting. The DT operates in a goal-conditioned and reward-conditioned format, where each input sequence includes a representation of the goal that, combined with the representation of the desired returns, guides the model in the generation stage. During training, a curriculum learning strategy was employed, training the model for two epochs on the first goal and switching to multi-target configuration for three more epochs for a total of five training epochs. We used a character-level tokenizer, trained on the ZINC-SMILES dataset, for molecular representation in all our experiments.

### 3.3 Metrics

We evaluate model performance using standard generative metrics (Validity, Novelty, Diversity) and specific objective targets: QED, pLogP, and TDC oracle binding affinities for DRD2 and JNK3. We also track the Success Rate (SR), the percentage of valid molecules meeting all objective targets within a 10% tolerance. To evaluate Pareto-front quality for multi-objective tasks, we report Hypervolume (HV) and the R2 indicator.

Validity, Novelty, and Diversity are essential for evaluating the generative capabilities of the models and serve as key indicators for poor performance. For instance, low Novelty often indicates that a model has overfitted to the training data. QED and pLogP serve as goal-specific optimization objectives, reflecting practical therapeutic and synthetic desirability. For evaluation, QED and pLogP RTG values were set to 0.95 and 4.5, respectively, representing high-end values, close to the upper bound, in the training data. The target RTGs for DRD2 and JNK3 were likewise set to prioritize high-affinity candidates based on the training data distributions. We sampled 5K molecules from each model, following pretraining where applicable, to compare generation capabilities without exploration, and again after optimization to assess performance post-fine-tuning. QED and pLogP scores were only computed for valid molecules within this set, so invalid sequences will not skew the results. This protocol enables a fair and consistent comparison across all baselines and our proposed method.

### 3.4 Experimental results

Table 1 and Fig. 2 present the performance of our M-GoRT method against baseline models. Only M-GoRT and the online-finetuned REINVENT variant generate molecule libraries that surpass the ZINC dataset and achieve high SR across both objectives. While both methods yield higher mean scores and lower standard deviations, M-GoRT also achieves a superior multi-objective SR score. The reduced variance of both methods indicates a concentration of generated molecules around optimal property values, as illustrated by property distributions in Fig. 2. M-GoRT’s advantage stems from its goal-conditioned return signals as further demonstrated by our ablation study, in contrast to REINVENT’s scalarized rewards, which limit balanced optimization.

**Table 1:** Results comparison for molecular generation methods evaluated on the ZINC dataset.^a^

Model	QED	pLogP	Validity (%)	Diversity (%)	Novelty (%)	SR (%)
ZINC dataset	0.73 ± 0.14	−0.65 ± 2.03	–	–	–	–
JTVAE	0.79 ± 0.15	−1.81 ± 2.23	**100**	47	47	0
MolDQN	0.72 ± 0.15	0.15 ± 1.56	**100**	99	99	0
DrugEx2 (pretrain)	0.74 ± 0.14	0.32 ± 1.79	**100**	**100**	**100**	0
DrugEx2 (RL)	0.79 ± 0.12	0.51 ± 2.21	**100**	**100**	**100**	1
REINVENT (pretrain)	0.73 ± 0.14	−0.52 ± 2.03	92	**100**	**100**	0
REINVENT (RL)	**0.90 ± 0.04**	2.71 ± 0.42	98	26	26	48
GDSS	0.66 ± 0.15	−3.59 ± 4.28	**100**	**100**	**100**	0
CDGS	0.74 ± 0.13	−1.62 ± 3.08	**100**	**100**	**100**	0
MolStitch	0.71 ± 0.16	1.96 ± 0.87	**100**	**100**	99	0
GPT-o4-mini	0.73 ± 0.14	−0.96 ± 2.27	81	96	96	0
M-GoRT	0.85 ± 0.06	**2.93 ± 0.41**	98	95	95	**57**

a For QED and pLogP both mean score and standard deviation are presented. Bold and underlined values denote the best and second-best performers, respectively.

To evaluate the generalization capabilities of M-GoRT, we extended our evaluation to a four-objective optimization task, incorporating biological activity targets for DRD2 and JNK3 evaluated using TDC oracles. As shown in Table 2, the increased complexity of the objective space significantly impacts all models. Notably, the SR for all evaluated methods dropped to zero, as no single generated molecule met the success thresholds across all four metrics simultaneously. This demonstrates the “curse of dimensionality” in molecular design, where the intersection of multiple independent pharmacological constraints creates a highly sparse region of valid chemical space.

**Table 2:** Performance comparison of selected high-performing models across an expanded multi-objective optimization task.^a^

Model	QED	pLogP	DRD2	JNK3	Validity (%)	Diversity (%)	Novelty (%)
ZINC dataset	0.73 ± 0.14	−0.65 ± 2.03	0.01 ± 0.04	0.02 ± 0.03	–	–	–
DrugEx2 (RL)	**0.74 ± 0.14**	−0.32 ± 1.71	0.03 ± 0.09	0.02 ± 0.03	**100**	**100**	**100**
REINVENT (RL)	**0.74 ± 0.09**	**2.69 ± 0.51**	**0.97 ± 0.06**	**0.38 ± 0.11**	99	21	21
MolStitch	0.66 ± 0.15	0.69 ± 1.57	0.35 ± 0.38	0.01 ± 0.02	**100**	**100**	99
M-GoRT	0.73 ± 0.12	2.16 ± 1.80	0.32 ± 0.34	0.02 ± 0.05	49	98	98

a In addition to QED and pLogP, models are evaluated on binding affinity scores for Dopamine Receptor D2 (DRD2) and c-Jun N-terminal Kinase-3 (JNK3) using Therapeutics Data Commons (TDC) oracles. Mean scores and standard deviations are reported. Bold and underlined values denote the best and second-best performers, respectively.

While MolDQN and DrugEx2 w\RL methods were able to outperform the dataset mean on at least one objective, they did so to a lesser extent and with higher variance. In the four-objective task, DrugEx2 w\RL fails to significantly shift the distribution of the biological targets compared to the ZINC baseline and exhibits a decrease in pLogP performance compared to the two-objective task, while QED remains stagnant. This suggests that traditional RL exploration struggles to navigate these high-dimensional targets effectively. Predictably, the pretrained versions of REINVENT and DrugEx2 (without RL) achieved similar results to the training set because, without their RL optimization components, these models learn to mimic the dataset’s property distribution, not to explore the chemical space for improved properties. MolStitch similarly struggles under the expanded regime, where it shows moderate success in improving DRD2 scores and maintains high pLogP, but its QED mean falls below the training set average, and it remains ineffective at optimizing for JNK3. These results highlight the challenge of maintaining general drug-likeness while simultaneously attempting to optimize for specific biological activities. MolStitch, similarly to the pretrained models, yields near-perfect validity, diversity, and novelty; however, unlike them, achieves higher score on the pLogP metric while QED remains slightly below dataset mean. The GPT-o4-mini baseline, despite high-temperature sampling, produces QED and pLogP means near ZINC and exhibits the lowest validity. This prompt-only approach yields good diversity/novelty but lacks the explicit reward signals needed for consistent property improvement. Notably, JTVAE, GDSS, and CDGS display lower pLogP scores than the training set. This may be due to the scalarized optimization, which prioritize the single composite score at the expense of individual property values.

As previously observed, methods relying on iterative online RL optimization such as MolDQN, DrugEx2, and REINVENT, achieve strong performance on one or both of the objectives that make up their optimization goal. REINVENT w\RL attains a marginally higher mean QED and an improved QED-SR relative to M-GoRT but loses marginally on the same metrics for pLogP. REINVENT’s gain in QED score does come at a pronounced cost in diversity and novelty, underscoring the tradeoff that our offline, goal-conditioned strategy successfully mitigates.

This tradeoff amplifies in the four-objective task (Table 2). While REINVENT maximizes DRD2 and pLogP, it suffers a catastrophic collapse in diversity and novelty, indicating the RL process converges on narrow, mode-seeking solutions. In contrast, M-GoRT achieves a balanced profile, shifting pLogP and DRD2 distributions while preserving baseline QED and high structural diversity avoiding the mode-seeking behavior of REINVENT. However, navigating these high-dimensional constraints increases conditioning density, which strains the decoder and reduces SMILES validity compared to the two-objective task.

While MolDQN and DrugEx2 w\RL do not suffer the same reductions in diversity and novelty metrics, they also yield lower objective means and SR, particularly with respect to pLogP and its SR and their property distribution is nearly unchanged compared to the distribution seen in ZINC dataset in Fig. 2. It is possible these results may be attributed to the default training horizon provided in the original implementation and that additional RL update steps would have yielded higher objective means, at the expense of novelty and diversity, but this could not be tested within our available compute budget.

In contrast to M-GoRT, baselines struggle with balanced joint optimization. JTVAE’s validity-first strategy severely diminishes novelty and diversity by repeatedly sampling a limited scaffold repertoire. Diffusion models (CDGS, GDSS) maintain diversity but fail to shift target property distributions (Fig. 2), lacking mechanisms to drive out-of-distribution sampling or mediate conflicting objectives. MolStitch achieves high pLogP but poor QED and SR, as its framework optimizes for diverse tradeoffs rather than joint maximization. Consequently, models like MolStitch, GPT-o4-mini, and REINVENT achieve higher Pareto-quality metrics (HV and R2, Table 3) by covering extreme, antagonistic tradeoffs (e.g. high-QED/low-pLogP). However, such extremes lack practical utility in drug discovery. Figure 3 demonstrates that M-GoRT instead concentrates generative capacity on the pharmacologically relevant “sweet spot” where both objectives are jointly maximized. In this high-performance subspace, M-GoRT’s frontier dominates MolStitch and GPT-o4-mini, rivaling only REINVENT. Thus, M-GoRT’s lower HV and R2 scores reflect strategic “high-quality area concentration” rather than optimization failure, trading unviable edge coverage for viable candidates (Fig. 1, available as supplementary data at *Bioinformatics* online).

**Figure 3:**
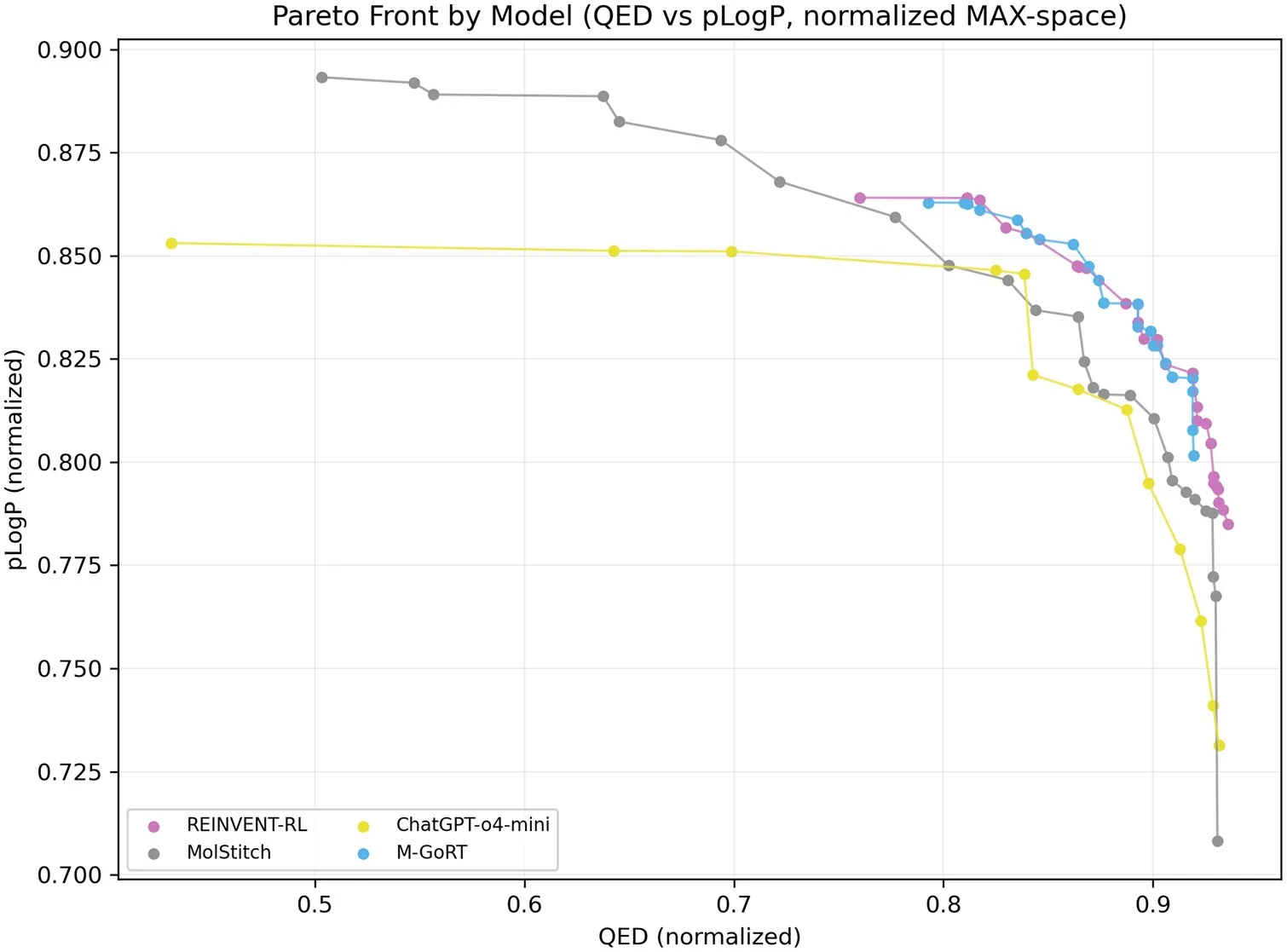
Pareto fronts for the top four models by hypervolume (HV) on the QED–pLogP plane (normalized MAX-space). Each polyline connects the non-dominated (Pareto-optimal) solutions among molecules generated by the corresponding model. Axes are normalized to a common MAX-space; points closer to the upper-right indicate better simultaneous optimization of QED and pLogP. Both M-GoRT and REINVENT form a frontier closest to the upper-right over a broad range, indicating the strongest tradeoff profile among the compared methods. HV is computed in the shared normalized space using a single reference point; see section 3.4 for details. A comprehensive Pareto-front figure including all baseline models is provided in the Supplementary Material, available as supplementary data at *Bioinformatics* online.

**Table 3:** Results comparison of Pareto metrics, Hypervolume, and R2 indicator for QED and pLogP metrics.^a^

Model	HV	R2
ZINC dataset	0.816	1.248
JTVAE	0.798	1.234
MolDQN	0.623	1.091
DrugEx2 (pretrain)^b^	0.824	1.255
DrugEx2 (RL)^b^	0.831	1.260
REINVENT (pretrain)	0.831	1.260
REINVENT (RL)	0.849	1.274
GDSS	0.764	1.208
CDGS	0.815	1.248
MolStitch	**0.855**	**1.280**
GPT-o4-mini	0.836	1.265
M-GoRT	0.834	1.263

a Bold text indicates the model that achieved the best results in each column, and underlined text indicates the second-best results.

b DrugEx2 results were generated using the graph-based variant.

When evaluating multi-objective SR, M-GoRT outperforms all baselines. A two-proportion *z*-test confirms that M-GoRT’s SR improvement over REINVENT is statistically significant (*P* < 10^−18^, 95% CI [6.9%,10.9%]). Overall, M-GoRT achieves simultaneous multi-property optimization while sustaining exploratory breadth, matching online RL efficacy using purely offline conditioning. Crucially, M-GoRT maintains competitive goal fidelity while retaining extensive structural variety, a balance that no alternative baseline achieves across both of the optimization objectives. Taken together, the comparative evidence underlines a consistent pattern: M-GoRT delivers strong, simultaneous optimization of multiple properties while sustaining high novelty and diversity, outperforming similar offline baselines that do not relay on exploration while competing with online RL pipelines that accomplish comparable results at the expense of exploratory breadth.

### 3.5 Ablation study

To evaluate the specific contributions of our architectural enhancements, we conducted an ablation study comparing M-GoRT with a standard single-target DT and a goal-conditioned DT, as detailed in Table 4.

**Table 4:** Results comparison of single-target DT, single-target goal-conditioned DT, and M-GoRT.^a^

Model	QED	pLogP	Validity (%)	Diversity (%)	Novelty (%)
Single target DT—QED	0.92 ± 0.04	−0.17 ± 1.20^b^	97	99	98
Single target DT—pLogP	0.40 ± 0.11^b^	4.33 ± 0.49	97	99	98
Goal-conditioned DT—QED	0.91 ± 0.05	0.05 ± 1.07^b^	98	99	98
Goal-conditioned DT—pLogP	0.43 ± 0.13^b^	3.96 ± 0.57	98	99	98
M-GoRT	0.85 ± 0.06	2.93 ± 0.41	98	95	95

a For QED and pLogP both mean score and standard deviation are presented.

b Off-target scores for properties the model did not directly optimize, e.g. pLogP for QED-targeted models.

Two single-target DT models were trained separately, optimizing exclusively for QED or pLogP, each conditioned on the respective scalar RTG during training and generation. As shown in Table 4, the QED-optimized model achieves the highest on-target QED. However, this specialization significantly compromises the off-target performance, with the off-target QED mean score notably lower than the ZINC dataset mean (Table 1). These findings underscore the inherent rigidity of single-objective training, limiting its ability to achieve balanced performance despite generating valid and novel molecules.

Goal-conditioned DT utilizes a shared reward block conditioned by explicit goal embeddings, training on interleaved trajectories for both objectives. While it approaches the single-target DT’s on-target performance, its off-target metrics significantly lag, indicating persistent gradient conflicts and insufficient multi-objective performance.

In contrast, M-GoRT assigns independent RTG blocks to each goal, effectively eliminating gradient conflict at the reward level and enabling simultaneous multi-objective optimization. Consequently, the model inherently balances QED and pLogP objectives without reporting separate off-target metrics. This approach incurs only a modest reduction relative to the best single-target scores, maintaining near-perfect validity, high diversity, and novelty.

To evaluate whether RTG conditioning functions as a continuous control signal, we varied the QED target at inference and examined the resulting score distributions as seen in Fig. 4. Increasing the RTG value systematically shifts the generated QED density toward higher values, while decreasing it shifts the density toward lower values. These resulting distributions are well-separated across settings while maintaining high validity. This monotonic, ordered response indicates that the goal-specific RTG blocks provide smooth, prompt-level control over property values using only the RTG value as conditioning, provided these values exist in the training data.

**Figure 4:**
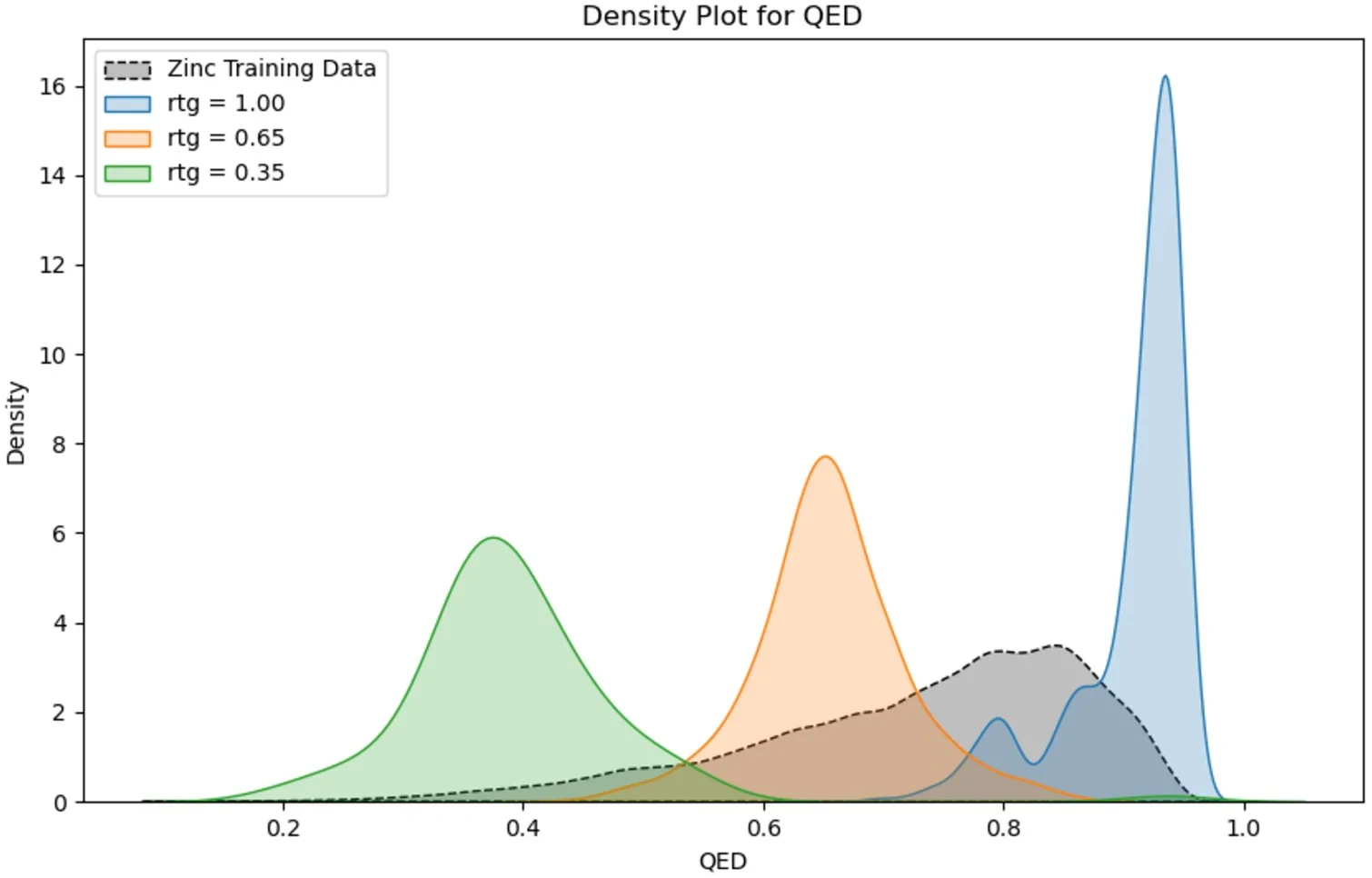
RTG conditioning governs objective values during generation. Density plots of QED for molecules sampled from single-target goal-conditioned decision transformer under three QED RTG prompts (1.00, 0.65, 0.35). The dashed black curve is the ZINC training distribution. Higher RTG shifts and concentrates the generated distribution toward higher QED, while lower RTG moves it to low-QED regions, demonstrating monotone, prompt-level control without retraining.

Overall, these comparisons illustrate that incorporating explicit goal-conditioning alone is insufficient for balanced multi-objective molecular generation. Decoupling reward pathways with separate goal-conditioned reward blocks is essential for effectively optimizing multiple conflicting objectives simultaneously.

## 4 Discussion

We proposed M-GoRT, which extends DTs with goal-specific RTG blocks to enable flexible, multi-objective molecule generation. Trained offline once, M-GoRT generates molecules satisfying various target combinations at inference, bypassing the costly retraining cycles of scalarized workflows. Our results show M-GoRT matches or surpasses strong baselines, shifting property distributions while maintaining high validity and diversity, and performing comparably to specialized single-target models. When scaling to high-dimensional tasks with complex biological targets (DRD2, JNK3), M-GoRT successfully shifts distributions without the catastrophic diversity collapse seen in online RL. While denser conditioning inevitably reduces SMILES validity, M-GoRT’s ability to maintain structural variety across conflicting objectives establishes it as a robust tool for complex lead optimization.

## Supplementary Material

btag731_Supplementary_Data
