# Supplementary material for: Multi-objective-conditioned-return-blocks decision transformer: a novel approach for multi-objective de novo drug design
Source: Bioinformatics. 2026 Oct 1;42(10):btag731. doi: 10.1093/bioinformatics/btag731 (PMC13674356; doi:10.1093/bioinformatics/btag731)
Supplement: Supplementary file 1 [file btag731_supplementary_data.pdf]

Multi-Goal-Conditioned-Return-Blocks Decision Transformer: A Novel Approach for Multi-Objective De Novo Drug Design-Supplementary Information

September 23, 2026

Table S1: Single-Objective Success-rate (SR) Results comparison for molecular generation methods. **Bold text** indicates the model that achieved the best results in each column and underlined text indicates the second best results.

Model	SR-QED[1]	SR-plogP[1]
JTVAE	59%	0%
MolDQN	11%	1%
DrugEx2 (Pretrain)[2]	40%	3%
DrugEx2 (RL)[2]	52%	10%
REINVENT (Pretrain)	21%	9%
REINVENT (RL)	**94%**	53%
GDSS	17%	2%
CDGS	37%	0%
MolStitch	80%	3%
GPT-o4-mini	36%	1%
M-GoRT	84%	**70%**

Listing 1: Prompt used to generate SMILES with GPT-o4-mini

```
You are an expert medicinal chemist with expertise in de-novo drug design
and molecular optimization.
Your task is to generate valid, novel, drug-like molecules that do not
exist in the ZINC database.

SPECIFIC REQUIREMENTS:
1. Generate 25 unique SMILES strings
2. Focus on maximizing QED (Quantitative Estimate of Drug-likeness)
3. Focus on maximizing pLogP (penalized LogP)
4. Ensure high structural diversity - avoid similar scaffolds
5. Create novel molecules that don't repeat existing SMILES in the ZINC database
6. All molecules must be synthetically feasible

OUTPUT FORMAT:
- Return ONLY the SMILES strings, one per line
- No explanations, no numbering, no additional text
- Ensure each SMILES is valid and canonical

Generate 25 valid, diverse, novel SMILES now:
```

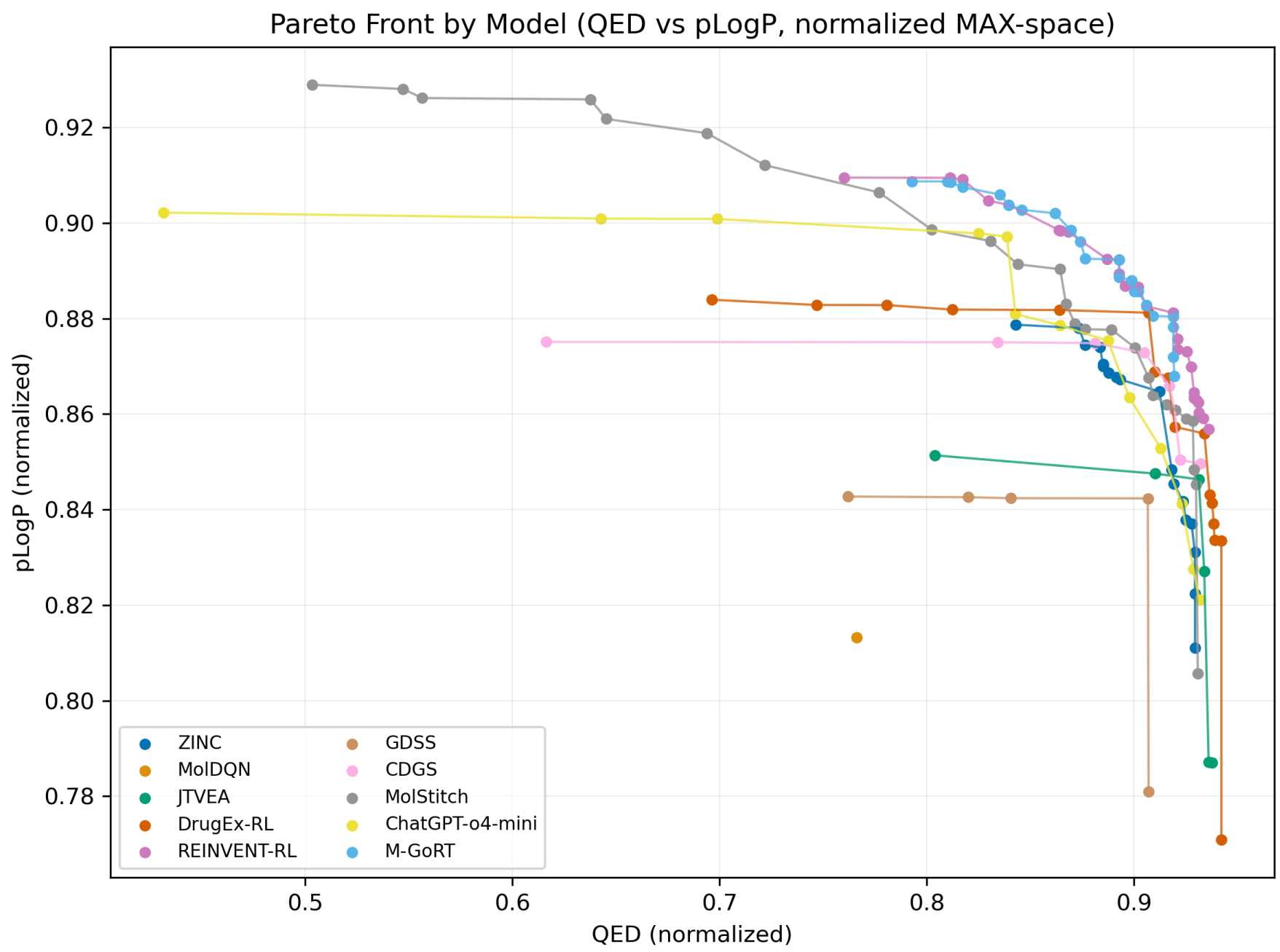

Figure S1: Comprehensive Pareto fronts across all evaluated models on the normalized QED–pLogP plane. Non-dominated sets are plotted per model after normalization to a common MAX-space and connected to visualize each method's trade-off curve. The global envelope highlights the best achievable region across methods. ZINC, MolDQN, JTVEA, DrugEx-RL, REINVENT-RL, GDSS, CDGS, MolStitch, GPT-o4-mini and M-GoRT are shown in blue, ochre, green, orange, purple, brown, pink, gray, gold and tea respectively.

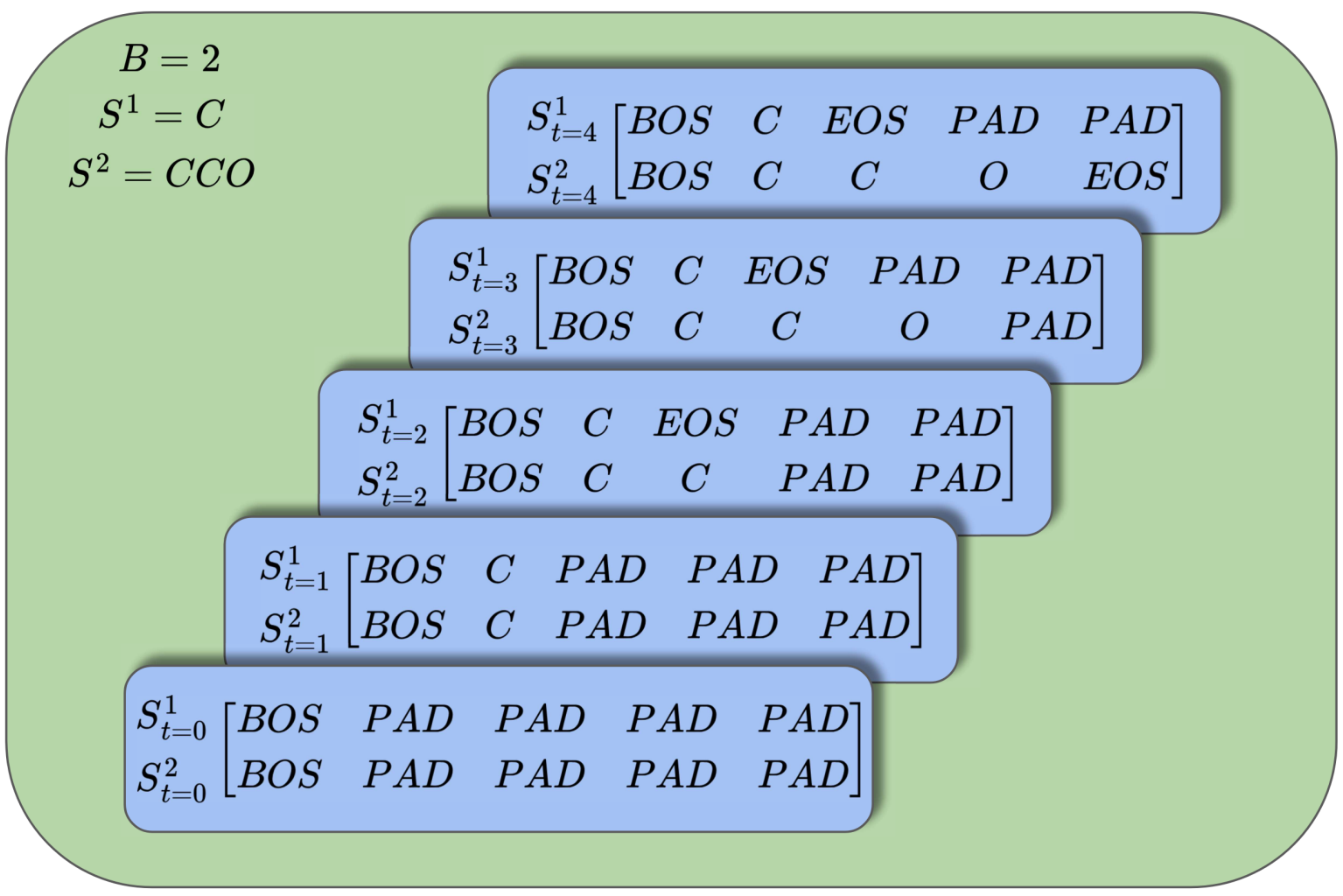

Figure S2: Illustration of a batched state tensor $\mathbf{S} \in N^{B\times n\times n}$ for two simple SMILES sequences ($B = 2$) of different lengths. Each matrix slice $\mathbf{S}_t^i$ corresponds to a training sample i at timestep t and is padded to the length of the longest sequence in the batch n. Rows begin with the `[BOS]` token, followed by the first t tokens of the sequence, and padded with `[PAD]` tokens. This structure enables the model to process batches of variable-length sequences uniformly during training and generation.
